# Detection of human papillomavirus DNA in formalin-fixed, paraffin-embedded squamous papillomas of the oral cavity

**DOI:** 10.4317/jced.55187

**Published:** 2018-10-01

**Authors:** Jack Daigrepont, Jennifer E. Cameron, Kelly L. Wright, Kitrina G. Cordell, Molly S. Rosebush

**Affiliations:** 1 DDS, Staff Dentist, SWLA Center for Health Services, Lake Charles, LA, USA; 2 PhD, Assistant Professor of Microbiology, Immunology & Parasitology, Louisiana State University, New Orleans, LA, USA; 3 MS, Predoctoral student, University of Florida College of Pharmacy, Gainesville, FL, USA; 4 DDS, MS, Associate Professor of Oral and Maxillofacial Pathology, Louisiana State University, New Orleans, LA, USA; 5 DDS, MS, Assistant Professor of Oral and Maxillofacial Pathology, Louisiana State University, New Orleans, LA, USA

## Abstract

**Background:**

Squamous papillomas are exophytic proliferations of surface oral epithelium. Human papillomavirus (HPV) infection is widely accepted as the etiology of squamous papillomas however the virus cannot be detected in a significant percentage of lesions.

**Material and Methods:**

Using polymerase chain reaction (PCR), we tested 35 formalin-fixed paraffin-embedded (FFPE) squamous papillomas for the presence of HPV DNA.

**Results:**

Six papillomas (17%) tested positive for HPV DNA; four contained HPV-6 and two contained HPV-11. Given that β–globin DNA was only identified in half of the samples, DNA degradation appears to have significantly impacted the results.

**Conclusions:**

The results likely represent an underestimation of the true number of HPV-positive specimens in our study. Potential explanations for HPV-negative squamous papillomas include transient HPV infection, failure of the experiment to detect HPV if present, or the possibility that some lesions may not result from HPV infection.

** Key words:**HPV, PCR, FFPE, papilloma, oral.

## Introduction

**Figure 1:**
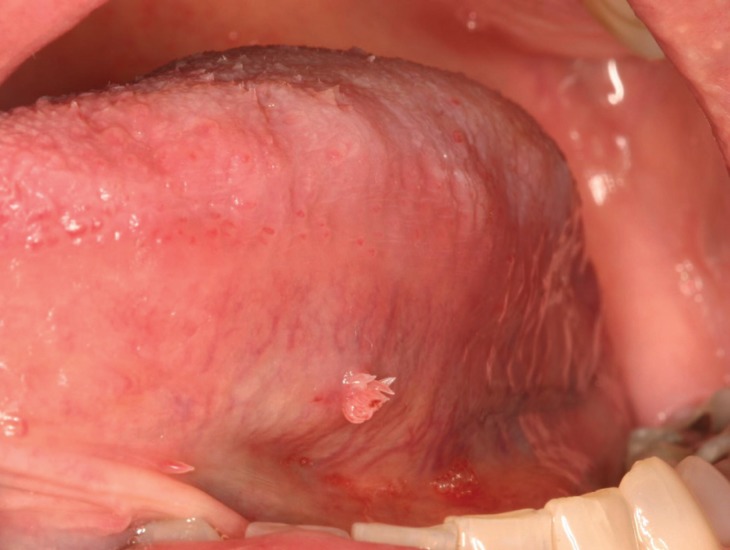
Clinical presentation of a squamous papilloma on the ventral tongue.

**Figure 2:**
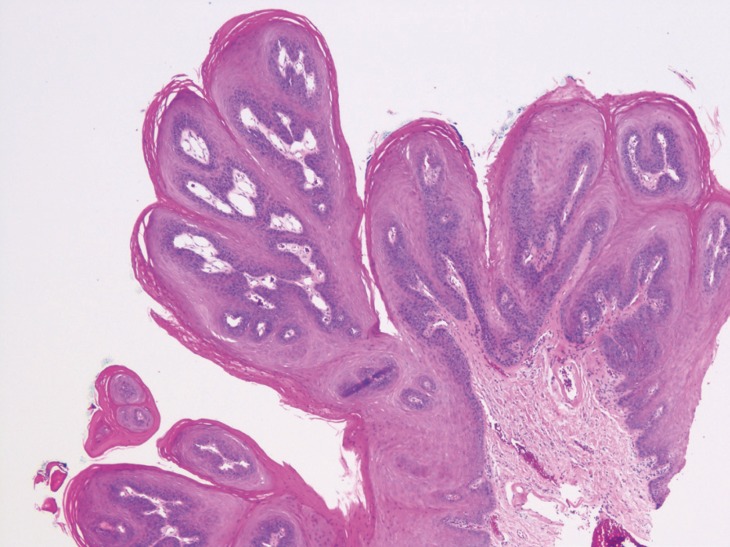
Histopathology of a squamous papilloma, low power.

Squamous papilloma represents the most common benign epithelial neoplasm of the oral cavity (1). The clinical presentation is a solitary, asymptomatic, exophytic lesion with either finger-like or cauliflower-like surface projections (Figs. 1,2). The color varies from pink to white depending on the amount of keratin produced. Squamous papillomas can occur at any age and in any oral location. The palate, tongue, and lips are most often affected (2).

The etiology of squamous papilloma has been attributed to infection with low-risk human papillomavirus (HPV) genotypes such as 6 and 11. HPV DNA is detected in an average of 34% of oral squamous papillomas however a wide range of detection rates have been reported using various experimental methods (3). HPV testing is not routinely performed on papillomas, therefore the precise contribution of HPV to the development of squamous papillomas is not fully understood. The possibility that some papillomas could have a non-viral cause is suggested by the inability to detect HPV DNA in a large proportion of lesions. In contrast, nearly all cases of oral condyloma acuminatum contain identifiable HPV (1,4) (5) and 86% of multifocal epithelial hyperplasia samples are HPV-positive (6).

Archived formalin-fixed paraffin-embedded (FFPE) tissue specimens represent a vast source of materials for use in research studies. FFPE tissues are the most common specimen stored in pathology laboratories. Analysis of these specimens can allow us to determine the prevalence of HPV genotypes in multiple clinical lesions. There is no gold standard or FDA-approved method for HPV detection in FFPE tissues (7). *In situ* hybridization (ISH) is labor intensive and lacks sensitivity, therefore polymerase chain reaction (PCR) has been the preferred method in recent studies (7,8). Large panels of HPV genotypes can be identified through the use of commercially-developed assays. In this study we sought to detect HPV DNA by PCR within FFPE oral squamous papillomas to determine prevalence of infection and specific viral genotypes.

## Material and Methods

Thirty-five FFPE squamous papillomas submitted to the LSU Oral Pathology Biopsy Service archives between 2014 and 2015 were selected. All specimens were reviewed microscopically by two oral pathologists (MR and KC) to confirm each was an ideal histopathologic representation of a squamous papilloma without significant overlapping features with condyloma acuminatum, verruca vulgaris, or multifocal epithelial hyperplasia. Six specimens diagnosed as fibroma were selected to serve as negative controls, and a previously confirmed HPV DNA-positive cervical biopsy specimen was used as a positive control. This study was approved by the Louisiana State University Health Sciences Center Institutional Review Board (IRB# 3974).

Using a microtome, five 10μm-thick sections were obtained from each specimen. Genomic DNA was extracted using the QIAmp FFPE Tissue Kit following the manufacturer’s instructions (Qiagen). The DNA extraction was performed immediately following tissue sectioning. Quantity of DNA was determined by spectrophotometry (NanoDrop). Quality of DNA was determined by polymerase chain reaction (PCR) for the human β-globin gene. If a sample contained detectable β-globin DNA, then it was assumed that HPV DNA should be intact and amplified during PCR. PCR was performed on the extracted specimen DNA using three separate HPV-specific primer sets: MY09/11, FAP59/64, and SPF1/2 according to published methods (9,10). The PCR products were visualized by agarose gel electrophoresis. For HPV DNA-positive specimens, genotype was determined by Roche Linear Array which detects the following 37 HPV genotypes: 6, 11, 16, 18, 26, 31, 33, 35, 39, 40, 42, 45, 51, 52, 53, 54, 55, 56, 58, 59, 61, 62, 64, 66, 67, 68, 69, 70, 71, 72, 73 (MM9), 81, 82 (MM4), 83 (MM7), 84 (MM8), IS39 and CP6108. Specimens testing positive for HPV were compared to specimens testing negative using a student’s t-test. A p-value of 0.05 was considered statistically significant.

## Results

The squamous papilloma specimens came from 20 males and 15 females (1.33 M:F). The average patient age was 52 years old (range 10-85). The papillomas were harvested from a variety of intraoral areas; soft palate and tongue were the two most frequent locations ( Table 1). The HPV DNA extraction results are summarized in  Table 2. Of the 35 squamous papillomas, 6 (17%) tested positive for HPV DNA; four specimens contained HPV-6 DNA and the other two contained HPV-11 DNA. Among the six specimens that tested positive for HPV DNA, there was 83.3% concordance across the three PCR assays, with one specimen testing positive by the FAP59/64 and SP1/2 PCR assays but negative on the MY09/11 PCR assay.

**Table 1:**
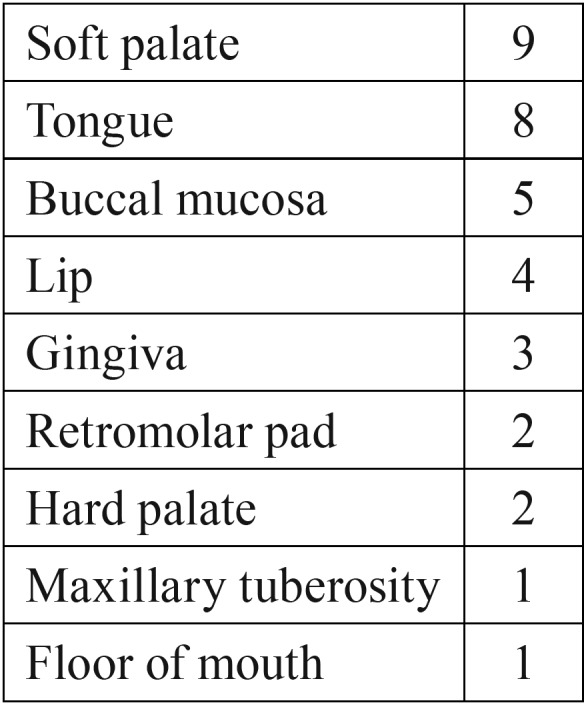
Clinical locations of 35 squamous papillomas.

**Table 2:**
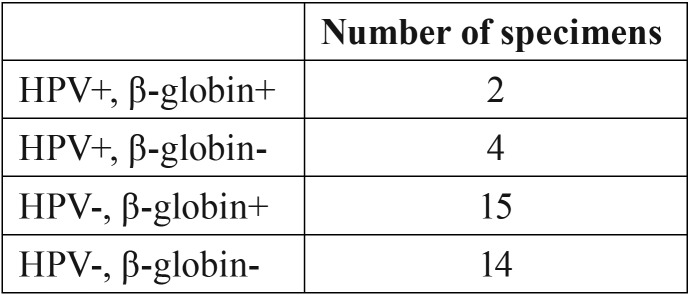
HPV DNA extraction results for 35 squamous papillomas.

The presence of β-globin DNA was detected in 17 of 35 papillomas (49%). Of the six HPV-positive samples, two (33%) were positive for β-globin. Two of the 6 fibromas were positive for β-globin and no fibromas were positive for HPV. When comparing HPV-positive and HPV-negative squamous papillomas in our sample, there were no statistically significant differences in DNA quality (*p*=.1167), DNA quantity (*p*=.095), or formalin fixation time (*p*=.736) ( Table 3).

**Table 3:**
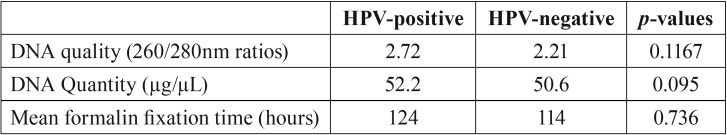
DNA quality, DNA quantity, and formalin fixation time for HPV-positive versus HPV-negative papillomas.

## Discussion

In this report, we detected HPV 6 or 11 in 6 out of 35 FFPE tissues diagnosed as oral squamous papillomas using a combination of HPV-specific PCR assays. Previous studies have identified HPV 6 and/or 11 in 13-35% of squamous papillomas by ISH (1,4,11) and in 48-68% of papillomas by PCR (5,12,13). The lower detection rates observed with ISH is likely due to inadequate sensitivity. PCR is a very sensitive technique and therefore higher rates of HPV detection have been seen in studies which utilized this method. A possible explanation for such a large variation in detection rate could be inclusion of cases of condyloma acuminatum in some studies (5). Additionally, small modifications to the experimental protocol can greatly influence amplification rates (14).

We detected HPV DNA in only 17% of the papillomas in our study which is much lower than prior studies using PCR. A possible explanation for this outcome is suboptimal DNA extraction from fixation. Fixation of pathology specimens and embedding in paraffin wax are essential steps in processing tissues for microscopic evaluation and long-term preservation. The most popular fixative, 10% neutral buffered formalin, stabilizes cells by the formation of protein crosslinks. Neutral buffered formalin has been identified as an acceptable fixative for the detection of HPV DNA by both ISH and PCR (8). In an experiment on cases of cervical cancer, Odida *et al.* found no statistically significant difference in HPV detection between freshly frozen samples and paraffin embedded tissue (15). HPV can reportedly be detected in FFPE samples for as many as 70 years post-fixation provided the target molecules are preserved (8). Unfortunately, over-fixation can cause excessive formation of protein crosslinks, reducing the effectiveness of DNA detection by PCR due to physical fragmentation of nucleic acids (8). Amplification of human cellular genes such as the β-globin gene is often used to determine the adequacy of extracted DNA for PCR amplification. If β–globin amplification is successful then HPV should be detected in that sample if present. β–globin DNA was only identified in 49% of our papillomas, suggesting that our samples suffered significant fragmentation of the DNA. Alternatively, the specimens may have contained analytes that significantly inhibited the enzymatic activity of the PCR reaction. These possibilities likely explain our low rate of HPV detection. Interestingly, however, the inability to detect β-globin did not preclude detection of HPV in our samples. Similar to our results, Park *et al.* identified HPV in both β–globin negative and β–globin positive samples (14). They postulated this could be due to a large quantity of HPV DNA present in the specimen or greater accessibility of HPV DNA compared to β–globin DNA. Other possible explanations could include pipetting error, differences in assay sensitivity or genomic variability of human β-globin sequences in primer target regions.

The amount of cross-linking has been reported as proportional to fixation time (8). Therefore, we examined the relationship between fixation time and the quality and quantity of DNA obtained from our specimens. We did not find any significant differences in DNA quality or quantity among our samples when accounting for length of time spent in formalin. Park et al. also found no decrease in β-globin amplification rate with specimens that had been stored for longer periods of time (14).

Investigators have used various approaches to mitigate the problem of DNA fragmentation. Kocjan *et al.* recommend an additional 1-hour incubation step for further removal of formalin crosslinks to improve amplification of HPV DNA fragments longer than 250 bp (8). Considering PCR assay design, Steinau *et al.* suggest that 450bp may represent the size limit for amplification of DNA from FFPE specimens (16). Amplification of shorter segments (<270 bp) of the HPV genome improves PCR sensitivity and is preferred for FFPE tissues (8). HPV detection rates have been shown to be significantly higher when targeting shorter PCR amplicons compared to larger ones because the shorter segments are likely to remain intact and be successfully amplified (14,17). In this study, we amplified a 65bp segment of the HPV L1 gene using the published primer pair designated SPF1/2 (9); however, this assay did not improve our HPV detection rates compared to the standard 450bp MY09/11 PCR assay typically used to amplify mucosal HPV genotypes.

Another possible explanation for the lack of detection of HPV in oral squamous papillomas is that the standard MY09/11 PCR assay was designed to target predominantly mucosal HPV genotypes commonly infecting the female genital tract. Studies have shown that oral specimens may contain HPV genotypes more commonly thought to infect the cutaneous epithelium (18-22). All three primers used in our study target a relatively conserved region of the L1 gene, which encodes the HPV major capsid protein. While MY09/11 primers detect a relatively broad spectrum of mucosal HPV genotypes, the FAP59/64 primer set (480bp) captures a broader spectrum of HPV genotypes, including cutaneous genotypes (10). Amplification with this primer set did not substantially improve our HPV detection rates over that of the MY09/11 PCR, consistent with the finding that our specimens contained HPV-6 or 11, the genotypes classically associated with oral squamous papilloma and genital warts.

The method of sample collection also appears to impact HPV detection, with much higher prevalence reported among exfoliated mucosal cells (by mouthwash or scraping) compared to biopsy specimens (23). Again, this is likely due to the need for formalin fixation of tissue that leads to poor quality DNA in comparison to that obtained from exfoliated cells. The reported prevalence of HPV DNA in normal oral mucosa ranges from 0-100% (23-25) but is estimated to be about 7% (26). Identification of HPV DNA in some squamous papillomas could simply represent oral mucosal carriage. However, HPV 16 is the most common genotype identified in normal oral mucosa (23,26) whereas genotypes 6 and 11 are the ones which have been identified in oral papillomas.

## Conclusion

Oral squamous papillomas have been attributed to low-risk HPV infections however viral DNA cannot be detected in a significant proportion of lesions. This contrasts with other papillary oral lesions such as condyloma acuminatum in which nearly all cases are HPV-positive. We consider the possibility that some papillomas may not be caused by HPV infection although there are multiple alternative explanations. Oral HPV infection could be transient in nature and therefore no longer detectable a certain time after having induced a clinical lesion. It is unlikely that the specimens contained genotypes different from the probes we used in our experiment. In agreement with previous reports, we detected no high-risk HPV genotypes within our papilloma specimens.

Technical challenges with DNA extraction from FFPE tissues are well known. Inadequate DNA extraction underestimates the prevalence of HPV. We believe this occurred in our study given the low percentage of β–globin positive samples. When we consider only the β–globin positive samples, 36% (6 of 17) contained HPV DNA; this may be a more accurate representation of true HPV prevalence in papillomas.
